# Bilateral hydroureteronephrosis with infection due to uterine prolapse

**DOI:** 10.1002/jgf2.295

**Published:** 2020-01-13

**Authors:** Koshi Ota, Yohei Sano, Akira Takasu

**Affiliations:** ^1^ Department of Emergency Medicine Osaka Medical College Takatsuki City Osaka Japan

## Abstract

Uterine prolapse could cause bilateral hydroureteronephrosis by kinking ureters.

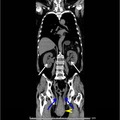

A 70‐year‐old woman presented to the emergency room (ER) with shivering and emesis that had not resolved for 1 week. She had a medical history of temporal arteritis and hepatitis B and was under medication with prednisolone (5 mg), tenofovir disoproxil fumarate (300 mg), and sulfamethoxazole trimethoprim once daily. Her delivery history was gravida 3 para 3 with spontaneous deliveries. Vital signs in the ER included tachypnea and tachycardia. Physical examination findings did not reveal any abnormality except for bilateral costovertebral angle tenderness. A nurse discovered uterine prolapse while collecting a urine specimen for culture. Computed tomography (CT) confirmed bilateral uterine prolapse and hydroureteronephrosis without bladder enlargement (Figures 1 and 2). Blood findings revealed mildly impaired renal function, increased C‐reactive protein and procalcitonin, and leukocytosis with neutrophilia. Urine cultures revealed *Escherichia coli*, but blood cultures were negative for organisms. Meropenem administered in the ER was de‐escalated to fosfomycin when the results of sensitivity tests became known. A gynecologist inserted the largest available pessary, CT showed hydroureteronephrosis resolved, and pyuria was also resolved clinically without other treatment thereafter. She was discharged without complications on hospital day 10. Although several reports have described uterine prolapse associated with bilateral hydroureteronephrosis,^1^, ^2^, ^3^ only a few have described CT findings of uterine prolapse associated with bilateral hydroureteronephrosis.^4^ Complete uterine prolapse could cause bilateral hydroureteronephrosis by kinking bilateral ureters.^5^ Patients should undergo a pelvic examination, and physicians should keep in mind urinary retention with postrenal renal failure when bilateral hydronephrosis is discovered by ultrasound or CT even if there is no complaint with pelvic organ prolapse, which should be treated adequately.

**Figure 1:**
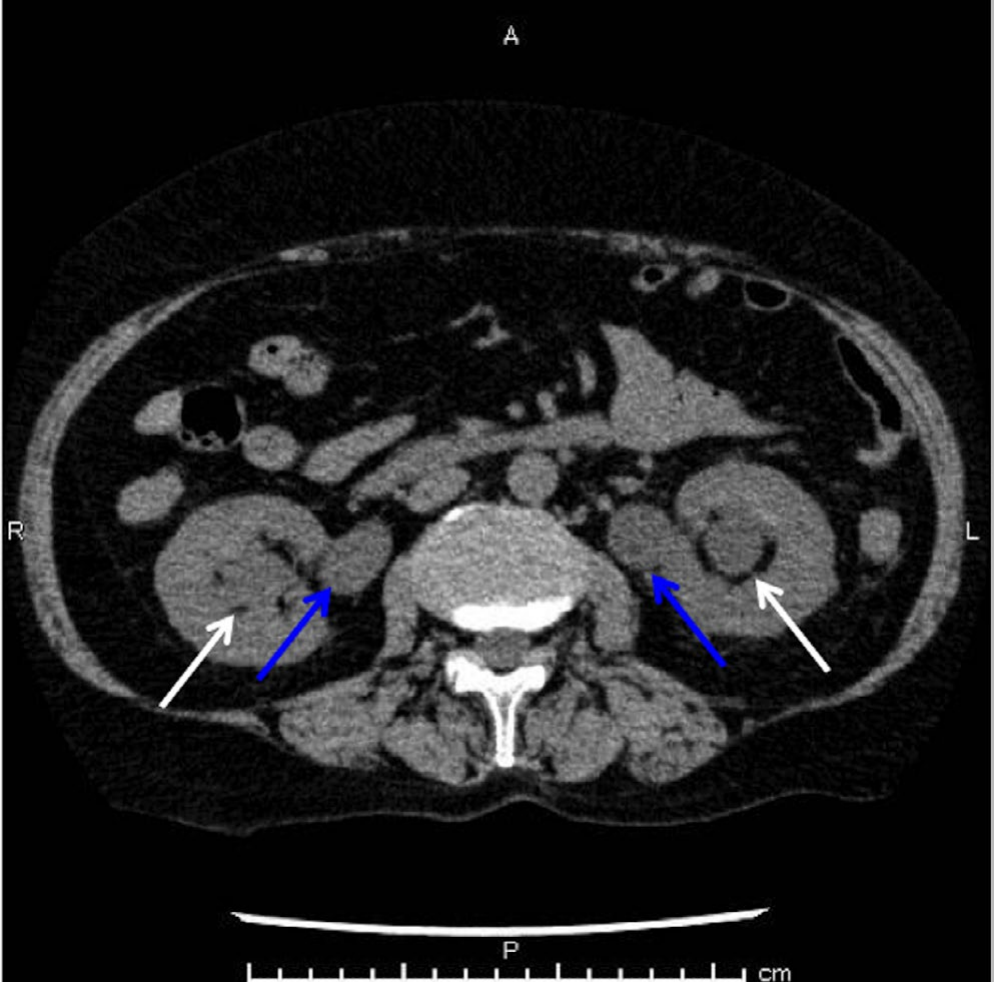
Computed tomography (CT) imaging findings. Transverse view shows bilateral hydroureteronephrosis (white arrow) and hydroureter (blue arrow)

**Figure 2:**
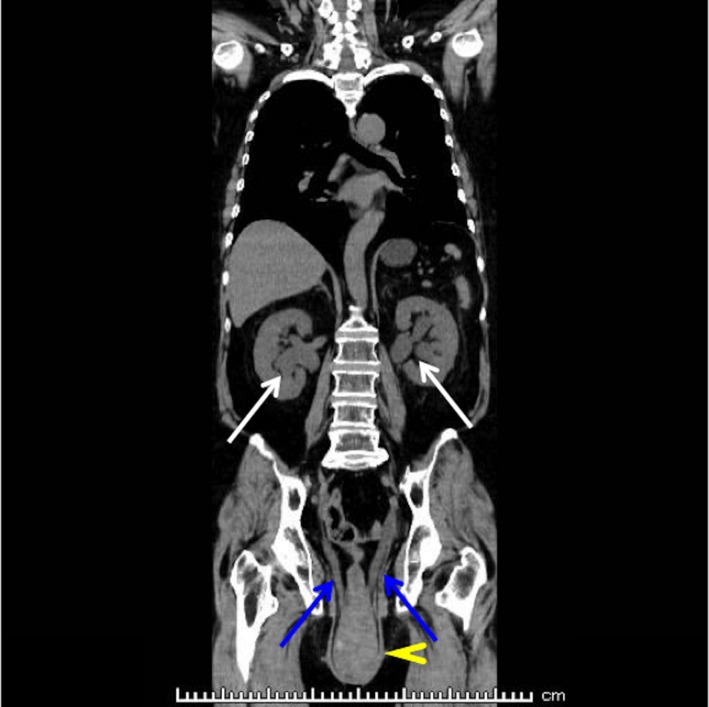
Computed tomography (CT) imaging findings. Coronal view shows total uterine prolapse (yellow arrowhead) with bilateral hydronephrosis (white arrow) and stretched hydroureter (blue arrow)

## CONFLICT OF INTERESTS

The authors have stated explicitly that there are no conflicts of interest in connection with this article.

## AUTHOR CONTRIBUTIONS

KO and AT prepared the article. KO collected the data. KO and YS managed the patient during admission. All authors have read and approved the final manuscript.

## ETHICS APPROVAL

Not applicable.

## CONSENT FOR PUBLICATION

Written informed consent was obtained from the patient and her husband for publication of this case report and the accompanying images.

## References

[1] Elkin M , Goldman SM , Meng CH . Ureteral obstruction in patients with uterine prolapse. Radiology. 1974;110(2):289–94. 4810138 10.1148/110.2.289

[2] Hadar H , Meiraz D . Total uterine prolapse causing hydroureteronephrosis. Surg Gynecol Obstet. 1980;150(5):711–4. 7368055

[3] Chuang FR , Lee CH , Chen CS , Weng HH , Wang IK . Bilateral moderate hydroureteronephrosis due to uterine prolapse: Two case reports and review of the literature. Ren Fail. 2003;25(5):879–84. 14575296 10.1081/jdi-120024303

[4] Bae EJ , Kang Y , Seo JW , Hwang K , Cho HS , Chang SH , et al. Obstructive uropathy by total uterine prolapse leading to end‐stage renal disease. Ren Fail. 2012;34(6):807–9. 22559223 10.3109/0886022X.2012.681533

[5] Dancz CE , Walker D , Thomas D , Özel B . Prevalence of hydronephrosis in women with advanced pelvic organ prolapse. Urology. 2015;86(2):250–4. 26194290 10.1016/j.urology.2015.05.005 PMC8605899

